# Individual and co-expression patterns of nerve growth factor and heme oxygenase-1 predict shorter survival of gastric carcinoma patients

**DOI:** 10.1186/s13000-017-0644-1

**Published:** 2017-07-05

**Authors:** Sang Jae Noh, Kyoung Min Kim, Kyu Yun Jang

**Affiliations:** 1 Department of Forensic Medicine, Chonbuk National University Medical School, Research Institute of Clinical Medicine of Chonbuk National University-Biomedical Research Institute of Chonbuk National University Hospital, 20, Geonji-ro, Deokjin-gu, Jeonju, Jeonbuk 54907 Republic of Korea; 2 Department of Pathology, Chonbuk National University Medical School, Research Institute of Clinical Medicine of Chonbuk National University-Biomedical Research Institute of Chonbuk National University Hospital and Research Institute for Endocrine Sciences, 20, Geonji-ro, Deokjin-gu, Jeonju, Jeonbuk 54907 Republic of Korea

**Keywords:** Nerve growth factor, Heme oxygenase-1, Prognosis, Stomach, Carcinoma

## Abstract

**Background:**

Nerve growth factor (NGF) is a neurotrophic factor which regulates cell development and proliferation. Recently, it has been suggested that NGF induces heme oxygenase-1 (HO1) expression, and that both NGF and HO1 are involved in the progression of malignant human tumors. However, exact roles of NGF and HO1 in tumorigenesis remain controversial. Therefore, we investigated the expression and correlation of NGF and HO1 in human gastric carcinoma tissues.

**Methods:**

We examined immunohistochemical expression of NGF and HO1 in 167 gastric carcinomas and compared with various prognostic clinicopathological factors.

**Results:**

The expression of NGF and HO1 was positive in 40% (67/167) and 51% (85/167) of cases, respectively, and their expression was significantly correlated with each other (*p* < 0.001). Individual expression patterns of NGF and HO1, and co-expression pattern of these two molecules were significantly associated with shorter survival by univariate analysis. HO1 expression (overall survival; *p* < 0.001, relapse-free survival; *p* = 0.002) and co-expression pattern of NGF and HO1 (overall survival; *p* = 0.002, relapse-free survival; *p* = 0.003) were independent poor prognostic indicators of gastric carcinoma patients by multivariate analysis.

**Conclusions:**

These results demonstrate that the individual and co-expression patterns of NGF and HO1 might be used as prognostic indicators for gastric carcinoma patients.

## Background

Nerve growth factor (NGF) is a member of neurotrophin family, and the effects of NGF are mediated by its binding to two types of NGF receptors (NGFR): high affinity tropomyosin receptor kinase A (TrkA) and low affinity p75 neurotrophin receptor (p75^NTR^) [1, 2]. However, in addition to the role of NGF in modulating proliferation, differentiation, and maturation of neuronal cells, NGF is a molecule of significant interest because of its involvement in the progression of various non-neuronal malignant tumors [1–3]. Binding of NGF to TrkA promotes cell survival, proliferation, and differentiation via phosphatidylinositol 3-kinase (PI3K)/AKT and Ras/MAPK signaling pathways [1]. In contrast, binding of NGF or precursor of NGF (proNGF) to p75^NTR^ could induce cell survival or could be pro-apoptotic [1]; therefore, as a consequence, there are conflicting reports for the role of NGF-related signaling in human malignant tumors. An oncogenic role of NGF has been supported by reports that NGF and NGFR participate in the progression of various human cancers, such as breast cancer [4, 5], ovarian cancer [6], pancreatic cancer [7], oral carcinoma [8, 9], and salivary gland cancer [10]. In addition, the expression patterns of NGF or TrkA were associated with poor prognosis of tongue cancer [9], pancreatic cancer [7], and breast cancer [5, 11]. However, a tumor-suppressive role of p75^NTR^ has been reported in pancreatic cancer [7] and colorectal cancer [12].

Heme oxygenase-1 (HO1) is a rate-limiting enzyme degrading heme into carbon monoxide, free iron, and bilirubin [13]. Because of the responsiveness of HO1 to variable stressors such as hyperthermia, hypoxia, heavy metals, irradiation, inflammatory cytokines, and oxidative stress, HO1 demonstrates anti-inflammatory, anti-apoptotic, and immunomodulatory effects [14, 15]. However, despite the protective role of HO1, it has been reported that HO1 could have a role in tumorigenesis. The expression of HO1 has been demonstrated to be anti-apoptotic [16, 17], pro-angiogenetic [18] and associated with increased invasiveness of cancer cells [19] and chemoresistance [20]. However, there are also controversial reports that found that HO1 inhibited the proliferation and metastatic capacity of the hepatocellular carcinoma [21] and that inhibition of HO1 enhanced the chemosensitivity for cisplatin in squamous cell carcinoma cells [22]. Therefore, the role of HO1 in cancer progression remains controversial.

Gastric cancer is one of the major causes of cancer death and is particularly common in low- and middle-income regions [23]. However, despite extensive studies on the impact of NGF and HO1 in major human cancers, the studies on NGF and HO1 in gastric carcinoma are limited. Moreover, their roles in human cancers have been controversial. Although, it has been reported that NGF expression is associated with poor prognosis of gastric carcinomas [24], other reports found that the expression of NGF gene is higher in normal gastric tissue than in gastric cancer tissue [25, 26]. Regarding HO1 in gastric carcinoma, HO1 was shown to be anti-apoptotic in gastric cancer cells [16] and microsatellite polymorphism with higher promoter activity of HO1 was associated with higher risk of gastric cancer in Japanese woman [27]. In addition, because there is a close relation between NGF and HO1 in malignant tumors [5, 28, 29], this study investigated the expression and correlation of NGF and HO1 in human gastric carcinoma tissues. Thereafter, we evaluated the expression of NGF and HO1 as a prognostic indicator for gastric carcinoma patients.

## Methods

### Gastric carcinoma patients and tissue samples

One hundred and sixty-seven cases of 177 cases of gastric carcinoma that we used in our previous study were the subject of this study [30]. The patients underwent operation between 1997 and 2005 at Chonbuk National University Hospital. However, among the 177 cases of gastric cancer, ten tissue microarray (TMA) cores were exhausted by our previous study. Therefore, 167 cases of gastric carcinoma were ultimately included in this study. Thereafter, we re-staged the 167 cases of gastric carcinoma according to the guidelines of the 8th edition of the of American Joint Committee on Cancer staging system [31] and reviewed them according to the WHO classification [32]. Clinical information for the patients was obtained by reviewing medical records. Pathologic variables were defined by reviewing pathologic reports and original histologic slides. In addition, 20 cases of noncancerous gastric mucosa obtained from the patients who were not related with gastric carcinoma were used for the evaluation of NGF and HO1 expression. This study received approval from the institutional review board of Chonbuk National University Hospital (IRB number, CUH 2013-04-011-001) on May 15, 2013, and the requirement for informed consent was waived.

### Gastric cancer cells and western blot

To validate antibodies for NGF and HO1 used in immunohistochemical staining, we performed western blotting analysis. Four human gastric cancer cell lines (MKN28, MKN45, KATO-III, and NCI-N87) were purchased from the Korean Cell line Bank (KCLB, Seoul, Korea) and cultured in RPMI1640 media. The cells were lysed with PRO-PREP Protein Extraction Solution (iNtRON Biotechnology Inc., Seongnam, Korea). Western blot was performed with primary antibodies for the NGF (Abcam, Cambridge, UK), HO1 (Enzo Life Sciences, Farmingdale, USA), and actin (Sigma, St. Louis, USA).

### Immunohistochemical staining and scoring for tissue microarrays

Immunohistochemical staining was performed on TMA tissue sections. The size of TMA core was 3.0 mm and contained one core per case because of the limited tumor thickness, especially in stage I gastric carcinomas. Microwave antigen retrieval procedure was performed with pH 6.0 Dako Target Retrieval Solution (DAKO, Glostrup, Denmark) for 20 min. Primary antibodies for NGF (Abcam, Cambridge, UK) and HO1 (Enzo Life Sciences, Farmingdale, USA) at a dilution of 1:200 have been used in immunohistochemical staining. The slides stained for NGF and HO1 were scored under a multi-viewing microscope by two pathologists (K.Y.J. and S.J.N.). The scoring performed with consensus of two investigators without clinicopathological information. The immunohistochemical staining slides were scored by the sum of the scale of staining intensity (no staining; 0, weak staining; 1, intermediate staining; 2, and strong staining; 3) and staining area (no staining cell; 0, 1%; 1, 2–10%; 2, 11–33%; 3, 34-66%; 4, and 67-100%; 5) [5, 33–35]. The score derived from the sum of the intensity scales and staining area scales ranged from zero to eight.

### Statistical analysis

Receiver operating characteristic curve analysis was performed to define positivity for the immunohistochemical expression of NGF and HO1. The cut-off points for the NGF- and HO1-positivity were determined at the point with the highest area under the curve to estimate survival of gastric carcinoma patients. The interest for the survival analysis was overall survival (OS) and relapse-free survival (RFS). The date of last follow-up was up to last contact or death of patients through June 2012. The event for the OS analysis was death of patients from gastric carcinoma. Alive patients at last follow-up date were treated as censored for OS analysis. The event for the RFS analysis was relapse or death from gastric carcinoma. Alive patients without relapse at last follow-up date were treated as censored for RFS analysis. Statistical analysis for the univariate and multivariate Cox proportional hazards regression analyses, Kaplan-Meier survival analysis, and Pearson’s chi-square test were performed using SPSS software (IBM, version 20.0, CA, USA). The *p*-values less than 0.05 were considered statistically significant.

## Results

### The expression of NGF and HO1 in human gastric carcinoma and its correlation with clinicopathologic factors

**Fig. 1:**
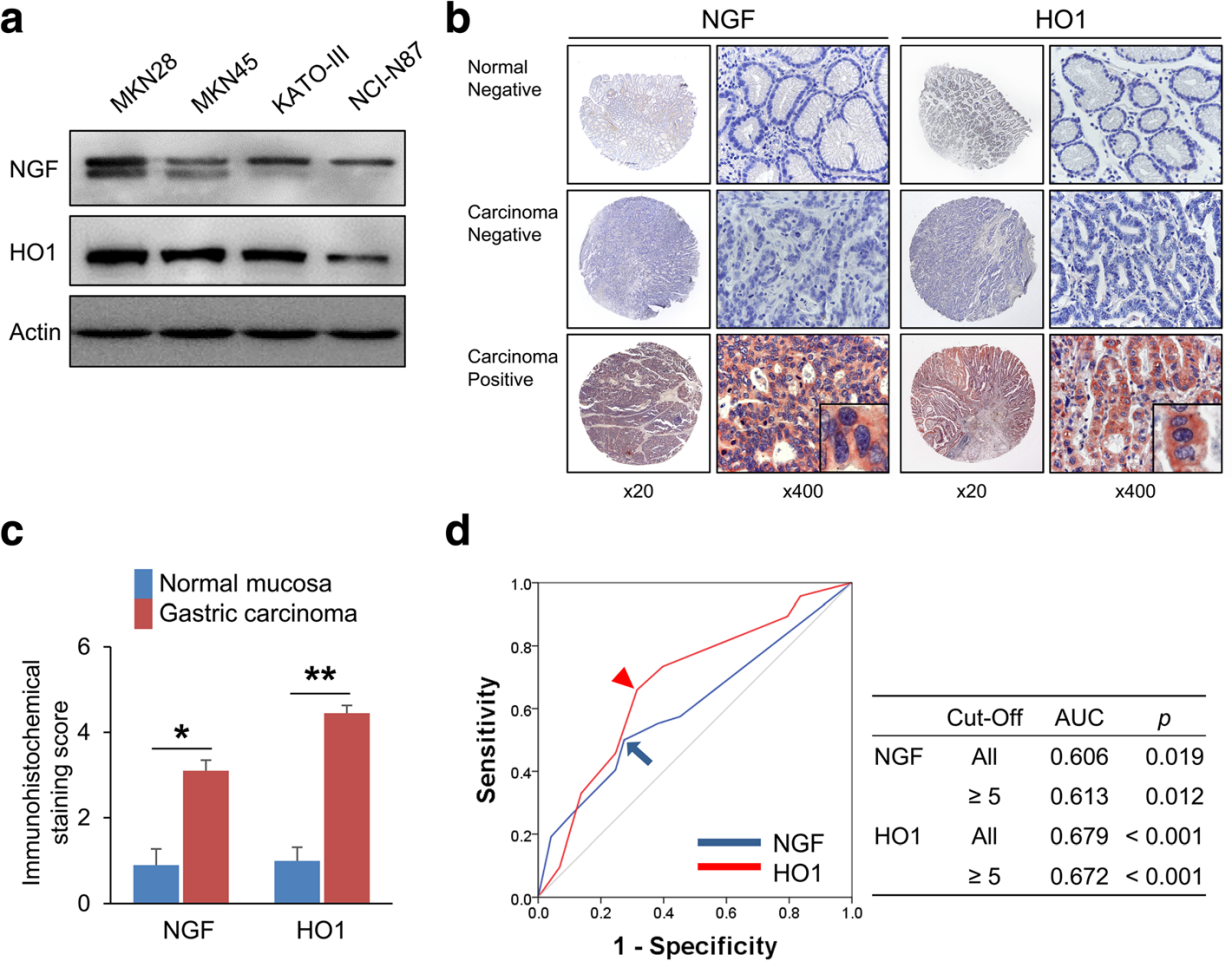
The expression of NGF and HO1 in human gastric cancer cells and human gastric carcinoma tissue. **a** Validation of the anti-NGF and anti-HO1 antibodies used in this study. The expressions of NGF and HO1 were seen in western blotting analysis in four gastric cancer cell lines (MKN28, MKN45, KATO-III, and NCI-N8). **b** Immunohistochemical expression of NGF and HO1 in normal gastric mucosa and gastric carcinoma. Original magnification: low-power images; ×20, high-power images; ×400. **c** Immunohistochemical staining scores of NGF and HO1 in normal gastric mucosa and gastric carcinoma. *; *p* < 0.05, **; *p* < 0.001. **d** Statistical analysis for the determination of immunohistochemical positivity for NGF (*arrow*) and HO1 (*arrow head*). The cut-off points were determined by receiver operating characteristic curve analysis at the highest area under the curve value for the estimation overall survival of gastric carcinoma patients

To validate anti-NGF and anti-HO1 antibodies, we performed western blot with four human gastric cancer cell lines. As we have shown in Fig. 1a, all gastric cancer cell lines expressed both NGF and HO1 and the expression patterns of NGF and HO1 were highest in MKN45 cells and lowest in NCI-N87 cells. Immunohistochemical expression patterns of NGF and HO1 in noncancerous gastric mucosa and gastric carcinoma shown in Fig. 1b. NGF and HO1 were primarily expressed in the cytoplasm of tumor cells. Even in the cases where NGF or HO1 were expressed in tumor cells, the non-neoplastic stromal cells did not express NGF or HO1 (Fig. 1b). Immunohistochemical expression scores for NGF (*p* = 0.003) and HO1 (*p* < 0.001) were significantly higher in gastric carcinomas (mean ± standard error: NGF; 3.1 ± 0.3, HO1; 4.5 ± 0.2) compared with normal gastric mucosa (mean ± standard error: NGF; 0.9 ± 0.4, HO1; 1.0 ± 0.3) (Fig. 1c). The cut-off points for the scores for the expression of NGF and HO1 were five for both stains (Fig. 1d). In addition, to verify the immunohistochemical negativity for NGF and HO1 in TMA sections, we performed additional immunohistochemical staining in full sections of 20 randomly selected cases from NGF-negative or HO1-negative subgroups in TMA analysis. In these cut-off values determined by TMA analysis, all cases immunostained for NGF (immunohistochemical staining score in 20 full sections: range; 0, mean; 0) and HO1 (immunohistochemical staining score in 20 full sections: range; 0–4, mean; 1.6) in full sections were also included in NGF-negative and HO1-negative subgroups. With this cut-off value, the expression of NGF and HO1 was positive in 40% (67 of 167 cases) and 51% (85 of 167 cases) of gastric carcinomas, respectively. There was significant association between NGF expression and older age of patients (*p* = 0.002), histological classification (*p* = 0.011), and Lauren classification (*p* = 0.010). The expression of HO1 was significantly associated with age (*p* = 0.012) and sex of patients (*p* < 0.001). In addition, the NGF-positivity was significantly associated with HO1-positivity in gastric carcinomas (*p* < 0.001) (Table 1).

**Table 1:** Clinicopathologic variables and the expression of NGF and HO1 in 167 gastric carcinomas

Characteristics		No.	NGF		HO1		NGF/HO1 expression pattern			
			Positive	*p*	Positive	*p*	−/−	−/+ or +/−	+/+	*p*
Age, years	<60	50	11 (22%)	0.002	18 (36%)	0.012	30 (60%)	11 (22%)	9 (18%)	<0.001
	≥60	117	56 (48%)		67 (57%)		30 (26%)	51 (44%)	36 (31%)	
Sex	Female	41	12 (29%)	0.103	34 (83%)	<0.001	7 (17%)	22 (54%)	12 (29%)	0.009
	Male	126	55 (44%)		51 (40%)		53 (42%)	40 (32%)	33 (26%)	
CEA^a^	Normal	107	40 (37%)	0.358	56 (52%)	0.232	39 (36%)	40 (37%)	28 (26%)	0.944
	Elevated	30	14 (47%)		12 (40%)		11 (37%)	12 (40%)	7 (23%)	
CA19-9^a^	Normal	121	47 (39%)	0.392	60 (50%)	0.975	44 (36%)	47 (39%)	30 (25%)	0.843
	Elevated	16	8 (50%)		8 (50%)		5 (31%)	6 (38%)	5 (31%)	
Tumor stage	I & II	77	27 (35%)	0.218	34 (46%)	0.107	33 (43%)	27 (35%)	17 (24%)	0.189
	III & IV	90	40 (44%)		51 (55%)		27 (30%)	35 (39%)	28 (31%)	
Tumor invasion	EGC	33	10 (30%)	0.199	16 (48%)	0.757	15 (45%)	10 (30%)	8 (24%)	0.435
	AGC	134	57 (43%)		69 (51%)		45 (34%)	52 (39%)	37 (28%)	
Lymph node metastasis	Absence	57	18 (32%)	0.105	26 (46%)	0.325	25 (44%)	20 (35%)	12 (21%)	0.256
	Presence	110	49 (45%)		59 (54%)		35 (32%)	42 (38%)	33 (30%)	
Venous invasion	Absence	137	53 (39%)	0.419	71 (52%)	0.609	51 (37%)	48 (35%)	38 (28%)	0.489
	Presence	30	14 (47%)		14 (47%)		9 (30%)	14 (47%)	7 (23%)	
WHO classification	Tubular	115	54 (47%)	0.011	65 (57%)	0.052	33 (29%)	45 (39%)	37 (32%)	0.010
	Signet ring cell	18	2 (11%)		8 (44%)		10 (56%)	6 (33%)	2 (11%)	
	Mucinous	17	4 (24%)		4 (24%)		9 (53%)	8 (47%)	0 (0%)	
	Mixed	13	5 (38%)		6 (46%)		6 (46%)	3 (23%)	4 (31%)	
	Papillary	2	2 (100%)		2 (100%)		0 (0%)	0 (0%)	2 (100%)	
	Neuroendocrine	2	0 (0%)		0 (0%)		2 (100%)	0 (0%)	0 (0%)	
Histologic grade^b^	WD	11	5 (45%)	0.326	7 (64%)	0.803	4 (36%)	2 (18%)	5 (45%)	0.433
	MD	65	35 (54%)		38 (58%)		15 (23%)	27 (42%)	23 (35%)	
	PD	41	16 (39%)		22 (54%)		14 (34%)	16 (39%)	11 (27%)	
Lauren classification	Intestinal	75	34 (45%)	0.010	44 (59%)	0.154	22 (29%)	28 (37%)	25 (33%)	0.226
	Diffuse	71	20 (28%)		33 (46%)		32 (45%)	25 (35%)	14 (20%)	
	Mixed	21	13 (62%)		8 (38%)		6 (29%)	9 (43%)	6 (29%)	
HO1	Negative	82	22 (27%)	<0.001						
	Positive	85	45 (53%)							

*Abbreviations*: *EGC* early gastric cancer, *AGC* advanced gastric cancer, *WD* well differentiated, *MD* moderately differentiated, *PD* poorly differentiated

^a^The data for the serum levels of CEA and CA19-9 were available in 137 and 137 patients, respectively

^b^Histologic grading was carried in tubular and papillary type carcinomas according to the grading system of the WHO histological classification of gastric tumors

### The expressions of NGF and HO1 were associated with shorter survival of gastric carcinoma patients by univariate analysis

**Fig. 2:**
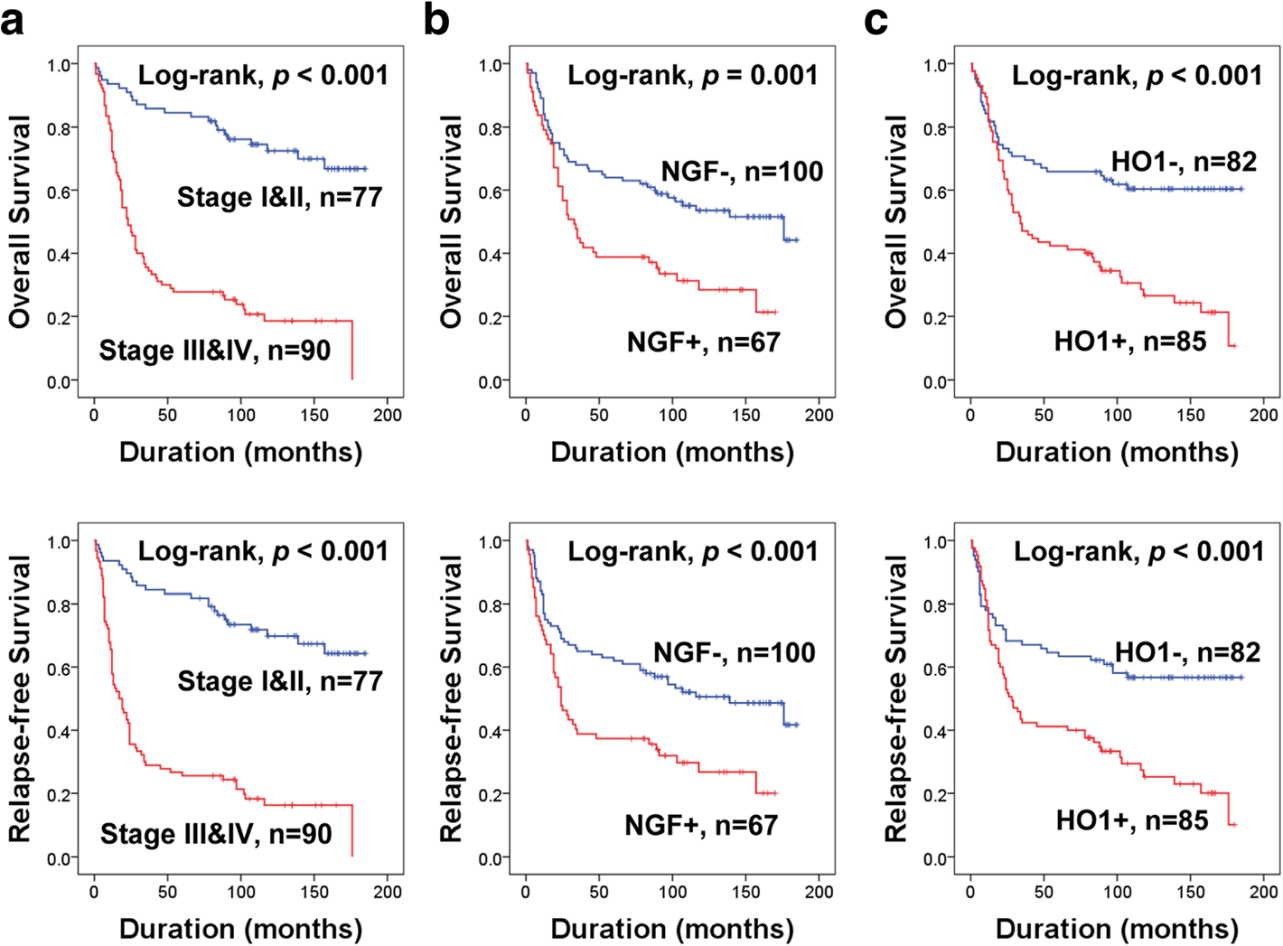
Kaplan-Meier survival analysis in 167 gastric carcinomas. The survival curves according to the tumor stage (**a**) and the expression of NGF (**b**) and HO1 (**c**)

Elevated preoperative serum levels of CEA and CA19-9, higher tumor stage, presence of lymph node metastasis and venous invasion, advanced gastric cancer type, higher histologic grade, and NGF- and HO1-positivity were significantly associated with both shorter OS and RFS of gastric carcinoma patients by univariate analysis (Table 2). The patients with tumors positive for NGF expression had a 1.943-fold [95% confidence interval (95% CI): 1.290-2.927, *p* < 0.001] greater risk of OS. NGF-positivity predicted a 1.932-fold (95% CI: 1.294-2.886, *p* < 0.001) greater risk of RFS. The expression of HO1 predicted a 2.358-fold (95% CI: 1.532-3.630, *p* < 0.001) greater risk of OS. HO1-positivity predicted a 2.185-fold (95% CI: 1.438-3.321, *p* < 0.001) greater risk of RFS of gastric carcinoma patients. The prognostic impact of tumor stage and the expressions of NGF and HO1 were subject to Kaplan-Meier survival analysis as shown in Fig. 2.

**Table 2:** Univariate Cox proportional hazards regression analysis for the survival of gastric carcinoma patients

Characteristics	No.	OS		RFS	
		HR (95% CI)	*p*	HR (95% CI)	*p*
Age, years, ≥ 60 (vs < 60)	117/167	1.232 (0.783-1.936)	0.367	1.169 (0.753-1.812)	0.487
Sex, male (vs female)	126/167	0.974 (0.608-1.559)	0.912	1.038 (0.650-1.657)	0.876
CEA^a^, elevated (vs normal)	30/137	2.087 (1.266-3.440)	0.004	1.948 (1.188-3.194)	0.008
CA19-9^a^, elevated (vs normal)	16/137	2.571 (1.407-4.696)	0.002	2.314 (1.272-4.211)	0.006
Tumor stage, III & IV (vs I & II)	90/167	5.309 (3.250-8.671)	<0.001	5.225 (3.252-8.393)	<0.001
Lymph node metastasis, presence (vs absence)	110/167	4.223 (2.448-7.286)	<0.001	4.311 (2.536-7.328)	<0.001
Venous invasion, presence (vs absence)	30/167	2.901 (1.827-4.607)	<0.001	2.789 (1.762-4.413)	<0.001
Tumor invasion, AGC (vs EGC)	134/167	4.105 (1.959-8.601)	<0.001	4.429 (2.118-9.262)	<0.001
Histologic grade^b^, WD	11/117	1	0.039	1	0.037
MD	65/117	2.713 (0.837-8.797)	0.096	2.832 (0.874-9.170)	0.083
PD	41/117	4.042 (1.229-13.294)	0.022	4.143 (1.262-13.602)	0.019
NGF, positive (vs negative)	68/167	1.943 (1.290-2.927)	0.001	1.932 (1.294-2.886)	0.001
HO1, positive (vs negative)	85/167	2.358 (1.532-3.630)	<0.001	2.185 (1.438-3.321)	<0.001
NGF/HO1 expression, −/−	60/167	1	<0.001	1	<0.001
−/+ or +/−	62/167	2.348 (1.365-4.037)	0.002	2.019 (1.203-3.389)	0.008
+/+	45/167	3.489 (1.995-6.101)	<0.001	3.218 (1.888-5.487)	<0.001

*Abbreviations*: *HR* hazard ratio, *EGC* early gastric cancer, *AGC* advanced gastric cancer

^a^The data for the serum levels of CEA and CA19-9 were available in 137 and 137 patients, respectively

^b^Histologic grading was carried in tubular and papillary type carcinomas according to the grading system of the WHO histological classification of gastric tumors

**Fig. 3:**
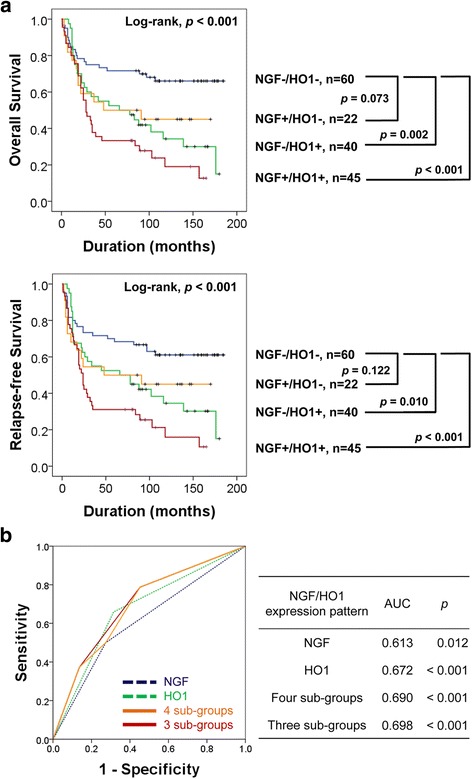
Kaplan-Meier survival analysis and statistical analysis according to the individual and co-expression pattern of NGF and HO1 in gastric carcinomas. **a** Gastric carcinomas were grouped into four sub-groups according to co-expression pattern of NGF and HO1 and subjected to Kaplan-Meier survival analysis for the overall survival and relapse-free survival. NGF^−^/HO1^−^, NGF-negative and HO1-negative. NGF^+^/HO1^−^, NGF-positive and HO1-negative. NGF^−^/HO1^+^, NGF-negative and HO1-positive. NGF^+^/HO1^+^, NGF-positive and HO1-positive. **b** Receiver operating characteristic curve analysis for the event of overall survival (death of patients) according to the NGF-positivity, HO1-positivity, and co-expression pattern of NGF and HO1 sub-grouping gastric carcinomas into four or three. The area under the curve is largest when the gastric carcinomas are divided to three sub-groups according to co-expression pattern of NGF and HO1 (NGF^−^/HO1^−^, NGF^+^/HO1^−^ or NGF^−^/HO1^+^, and NGF^+^/HO1^+^)

**Fig. 4:**
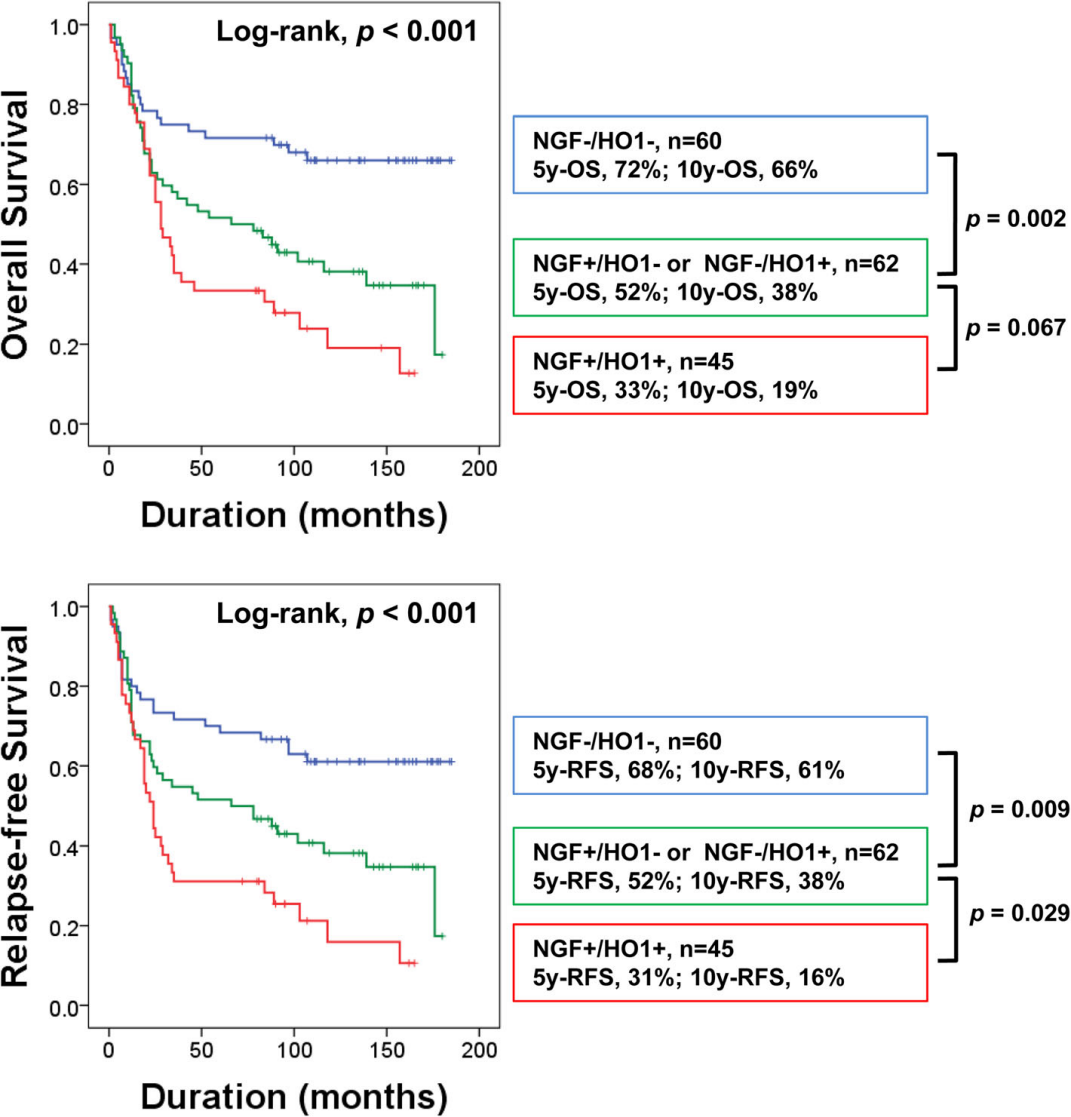
Kaplan-Meier survival analysis according to co-expression patterns of NGF and HO1 in 167 gastric carcinomas. Gastric carcinomas were grouped into three sub-groups according to the co-expression pattern of NGF and HO1. NGF^−^/HO1^−^, NGF-negative and HO1-negative. NGF^−^/HO1^−^, NGF-negative and HO1-negative. NGF^+^/HO1^−^, NGF-positive and HO1-negative. NGF^−^/HO1^+^, NGF-negative and HO1-positive. NGF^+^/HO1^+^, NGF-positive and HO1-positive. 5y-OS, overall survival rate at five-years. 10y-OS, overall survival rate at ten-years. 5y-RFS, relapse-free survival rate at five-years. 10y-RFS, relapse-free survival rate at ten-years

Based on the correlation between NGF and HO1 in previous reports [28, 29] and the significant association between NGF and HO1 expression in our results, we evaluated the prognostic significance of co-expression patterns of NGF and HO1. First, we grouped gastric carcinomas into four-groups according to the expression types of NGF and HO1 (NGF^−^/HO1^−^, NGF^+^/HO1^−^, NGF^−^/HO1^+^, and NGF^+^/HO1^+^) and performed a Kaplan-Meier survival analysis (Fig. 3a). Based on these results, we re-grouped the NGF/HO1 expression pattern into three sub-groups (NGF^−^/HO1^−^, NGF^+^/HO1^−^ or NGF^−^/HO1^+^, and NGF^+^/HO1^+^). Thereafter, we compared the prediction of death of gastric carcinoma patients with the individual or co-expression patterns of NGF and HO1 via receiver operating characteristic curve analysis. Three types of sub-groups of gastric carcinoma patients were grouped according to the largest area under the curve values for NGF and HO1 expression (Fig. 3b). This type of co-expression pattern of NGF and HO1 was significantly associated with shorter OS (overall *p* < 0.001) and RFS (overall *p* < 0.001) by univariate analysis (Table 2). Gastric carcinoma patients with a NGF^+^/HO1^+^ expression pattern showed only a 33% five-year OS rate and a 19% ten-year OS rate. In contrast, the patients with a NGF^−^/HO1^−^ pattern showed a 72% fiver-year OS rate and a 66% ten-year OS rate. The survival rate of ‘NGF^+^/HO1^−^ or NGF^−^/HO1^+^’ sub-group was intermediate to the NGF^−^/HO1^−^ and NGF^+^/HO1^+^ sub-groups (five-year OS rate; 52%, ten-year OS rate; 38%) (Fig. 4). The prognostic impact of the NGF/HO1 expression pattern for the estimation of RFS was consistent with the results of analysis for the OS (Table 2 and Fig. 4).

### The expression of HO1 and co-expression pattern of NGF and HO1 are independent indicators of poor prognosis of gastric carcinoma patients by multivariate analysis

The variables predicting survival of gastric carcinoma patients in univariate OS and RFS analysis were included in the multivariate analysis. Tumor stage, venous invasion, and HO1 expression were independent indicators of poor prognosis of OS and RFS (Table 3, model 1). The expression of HO1 in gastric carcinomas predicted a 2.382-fold (95% CI; 1.464-3.876, *p* < 0.001) greater risk of OS and a 2.099-fold (95% CI; 1.312-3.356) greater risk of RFS. In addition, when we included the combined NGF/HO1 expression pattern instead of individual expression of NGF and HO1 in multivariate analysis, tumor stage (OS; *p* < 0.001, RFS; *p* < 0.001), venous invasion (OS; *p* = 0.005, RFS; *p* = 0.007), and the co-expression pattern of NGF and HO1 (OS; overall *p* = 0.002, RFS; overall *p* = 0.003) were independent indicators of poor prognosis of gastric carcinoma patients (Table 3).

**Table 3:** Multivariate Cox regression analysis for the survival of gastric carcinoma patients

Characteristics	OS		RFS	
	HR (95% CI)	*p*	HR (95% CI)	*p*
Model 1^a^				
Tumor stage, III & IV (vs I & II)	4.226 (2.406-7.422)	<0.001	4.134 (2.414-7.082)	<0.001
Venous invasion, presence (vs absence)	2.129 (1.249-3.629)	0.006	1.959 (1.156-3.317)	0.012
HO1, positive (vs negative)	2.382 (1.464-3.876)	<0.001	2.099 (1.312-3.356)	0.002
Model 2^b^				
Tumor stage, III & IV (vs I and II)	4.373 (2.482-7.706)	<0.001	4.203 (2.449-7.214)	<0.001
Venous invasion, presence (vs absence)	2.147 (1.252-3.681)	0.005	2.104 (1.231-3.597)	0.007
NGF/HO1 expression, −/−	1	0.002	1	0.003
−/+ or +/−	2.222 (1.230-4.014)	0.008	1.888 (1.074-3.320)	0.038
+/+^+^	3.040 (1.628-5.677)	<0.001	2.818 (1.552-5.116)	0.001

*Abbreviations*: *HR* hazard ratio

^a^Variables considered in the Model 1 were the pretreatment serum level of CEA and CA19-9, histologic grade, tumor stage, tumor invasion (EGC versus AGC), lymph node metastasis, venous invasion, and expression of NGF and HO1

^b^Variables considered in the Model 2 were the pretreatment serum level of CEA and CA19-9, histologic grade, tumor stage, tumor invasion, lymph node metastasis, venous invasion, and co-expression pattern of NGF and HO1

## Discussion

**Fig. 5:**
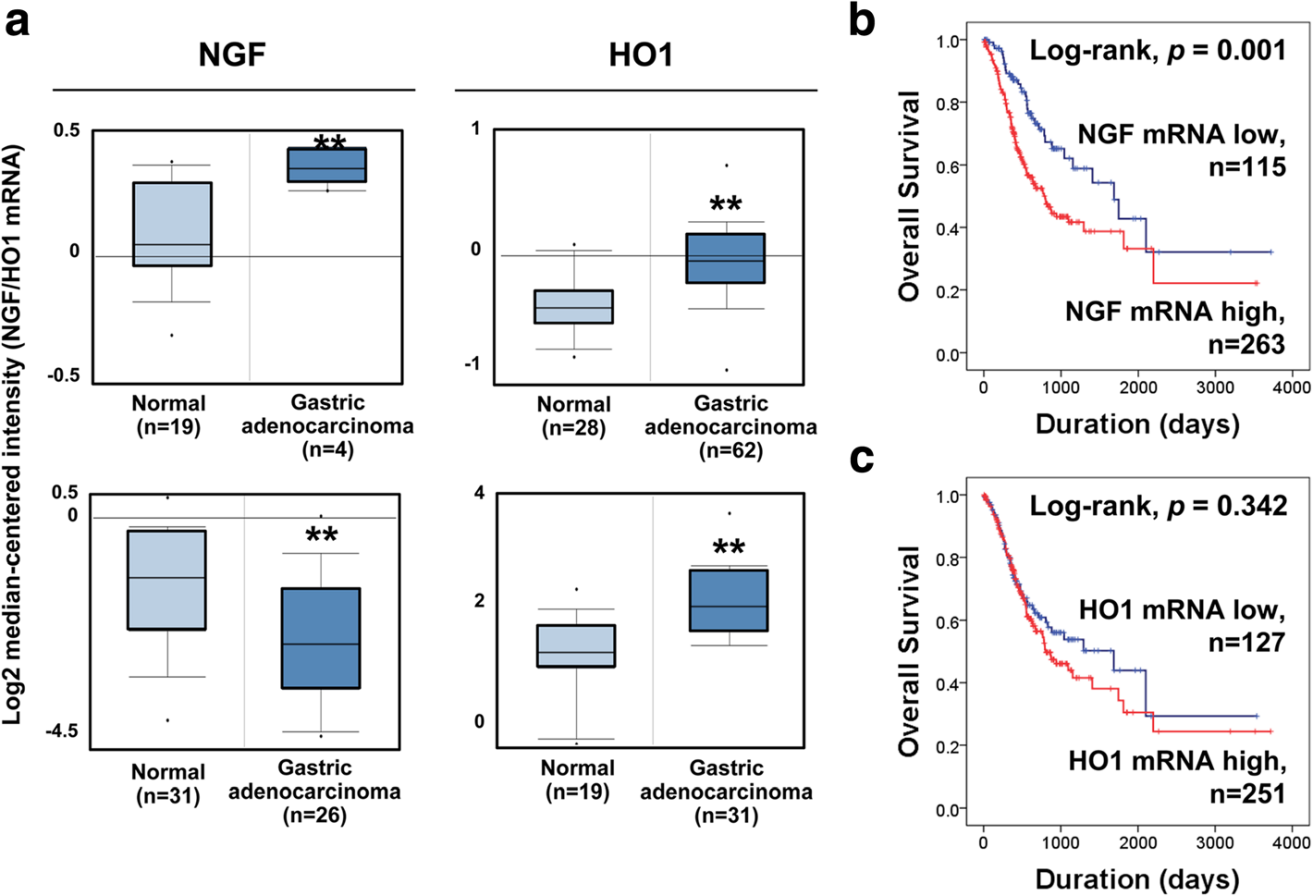
The mRNA expression of NGF and HO1 in normal gastric tissue and gastric carcinoma and their prognostic impact. **a** The expression of mRNA of NGF and HO1 in normal gastric mucosa and gastric carcinoma obtained from the Oncomine database (https://www.oncomine.org. Accessed 22 April 2017). **b** Kaplan-Meier survival analysis according to the mRNA expression of NGF and HO1 in gastric carcinomas. The survival data obtained by a search of the OncoLnc database (http://www.oncolnc.org. Accessed 22 April 2017)

In this study, we demonstrate that substantial cases of gastric carcinomas express NGF and HO1, and their expression patterns are significantly associated with each other. In addition, the expression patterns of NGF and HO1 were higher in gastric carcinomas compared with normal gastric mucosa (Fig. 1c). Similarly, a search of the Oncomine database (search condition: threshold *p* value; *p* < 0.001, threshold gene rank; top 10% gene, data type; mRNA) showed higher expression of HO1 mRNA in gastric carcinomas compared with normal gastric tissue (Fig. 5a) although the expression of NGF mRNA was controversial [36]. Especially, the expression patterns of NGF and HO1 in gastric carcinomas were significantly associated with shorter OS and RFS. These findings suggest that NGF-HO1 related pathways might have a role in the progression of gastric carcinomas. Similarly, higher expression of NGF mRNA was significantly associated with shorter OS of gastric cancer patients in search of OncoLnc database (Log-rank, *p* < 0.001) (Fig. 5b) [37]. In line with our results, the expression patterns of NGF and HO1 were associated with advanced clinicopathologic factors of breast carcinomas, and were independent indicators of poor prognosis of breast carcinoma patients [5]. In addition to the expression of NGF, the expression of NGFR was also associated with progressive factors of breast carcinomas, such as HER2 expression, estrogen receptor negativity, and higher histologic grade [38].

Regarding the role of the NGF signaling pathway in tumorigenesis, an association between the expression of NGFR TrkA and p75^NTR^ has been reported in various types of cancers. NGF is involved in the proliferation of breast cancer cells through the NGFR TrkA-MAPK pathway and it prevents apoptosis by activating the NGFR p75^NTR^-NFκB pathway [39]. Overexpression of TrkA promoted growth and metastatic potential of xenografted breast cancer cells by activating the MEK and PI3K pathways and enhancing resistance to anoikis [4]. In pancreatic cancer, mRNA expression of TrkA was associated with an aggressive tumor phenotype with perineural invasion and a higher degree of pain [7]. However, this study found mRNA expression of p75^NTR^ to be an indicator of favorable prognosis [7]. When considering NGF-NGFR as a therapeutic target of human malignant tumors, NGF might confer chemoresistance of cancer cells due to the role of NGF in the resistance to oxidative stress and apoptosis [28, 29]. In addition, proNGF produced by cancer cells is involved in the invasiveness of breast cancer cells [40]. Therefore, inhibition of proNGF, NGF, or NGFR has been proposed as therapeutic stratagems for human malignant tumors [40–42]. Small interfering RNA against NGF or proNGF, anti-NGF antibodies, and inhibitors of NGFR were pro-apoptotic and anti-proliferative for cancer cells and inhibited tumor growth and invasiveness [40–42]. Moreover, since there is a possibility that NGF selectively influences the proliferation of breast cancer cells but not normal breast epithelial cells, NGF might be an optimal therapeutic target for particular cancer types [43]; the effect of NGF on cancer cells differs according to the expression status of TrkA and/or p75^NTR^ and varies with the use of chemotherapeutic agents [44]. Despite some limitations, these finding suggest that the NGF pathway could be a useful therapeutic target for the treatment of malignant human tumors. Although, the study for the NGF as a therapeutic target of gastric carcinoma is limited, our results suggest that NGF-related pathways are important in the progression of gastric carcinomas by presenting NGF expression as a prognostic marker of gastric carcinoma. Similarly, a recent report has shown that the immunohistochemical expression of NGFR TrkA was associated with advanced clinicopathological factors of gastric carcinomas, such as lymph node metastasis and distant metastasis, and predicted shorter disease-specific survival and RFS [24]. However, there are also reports that seem to contradict these. The mRNA levels of NGF and TrkA were lower in gastric cancer compared with normal gastric mucosa [25]. Therefore, further study is needed to clear the role of NGF in gastric carcinogenesis.

In our study, the expression patterns of NGF and HO1 in gastric cancer tissue were significantly associated with each other. 45 of 67 (67%) cases of NGF-positive gastric carcinomas were positive for HO1 and 60 of 100 NGF-negative cases were also negative for HO1. When we compared the immunohistochemical staining scores between the expression of NGF and HO1, the expression of NGF significantly correlated with the expression of HO1 (Pearson’s correlation coefficient; *r* = 0.329, *p* < 0.001). This result might be related to the fact that NGF induces expression of HO1. NGF stimulates gene expression of HO1 and causes resistance to oxidative stress through MEK- and a PI3K-dependent mechanisms [28, 29]. The relationship between NGF and HO1 in the resistance to stress might be a mechanism for the progression of malignant human tumors. Supportive of this hypothesis, we found that the expressions of both NGF and HO1 were significantly associated with shorter survival of gastric carcinoma patients. Especially, the individualized HO1 expression and co-expression pattern of NGF and HO1 were independent indicators of poor prognosis of gastric carcinoma. The NGF^+^/HO1^+^ subgroup showed the shortest survival and the NGF^−^/HO1^−^ subgroup of gastric carcinoma patients had the longest survival time. We published similar results in breast carcinomas. The expression of NGF, HO1, and the combined NGF/HO1 expression patterns were independent indicators of poor prognosis of breast carcinomas [5]. Similarly, the expression of HO1 predicted shorter survival of non-small cell lung cancer patients [19]. These findings suggest that evaluation of individual and co-expression pattern of NGF and HO1 in human cancer tissue might be helpful for prediction of outcome. In addition, higher expression of HO1 was associated with resistance to apoptosis of gastric cancer cells [16] and increased invasiveness of non-small cell lung cancers [19]. HO1 also promotes tumor progression by inducing angiogenesis in pancreatic cancer [45] and urothelial carcinoma [18]. Moreover, therapeutic efficacy of the inhibition of HO1 was presented in pancreatic cancer cells. The mRNA and protein levels of HO1 were higher in pancreatic cancer cells compared with normal pancreatic tissue and suppression of HO1 with siRNA inhibited proliferation and sensitize tumors to chemo- and radio-therapy [46]. However, there are also discrepancies in reports on the role of HO1 in tumorigenesis. In a small study that analyzed 55 colorectal cancer patients, HO1 expression was associated with fewer lymph node metastases and favorable survival of patients [47]. Inhibition of HO1 with siRNA increased the proliferation and invasiveness of androgen-sensitive prostate cancer cells and breast cancer cells [48, 49]. In addition, overexpression of HO1 inhibited proliferation and invasion via down-regulation of MMP9 in pancreatic and breast cancer cells [48, 49]. These conflicting results might be related with the varied roles of the NGF-HO1 pathway in the resistance to stress. As we have seen with the expression of DNA damage response molecules, resistance to the stresses could be tumor suppressive or induce resistance to anti-cancer therapy [33, 35, 50]. Defects in BRCA1/2 promote the early development of breast and ovarian carcinomas, but also make cancer cells sensitive to DNA damage-inducing anti-cancer therapy and anti-PARP therapy [50, 51]. When stress-resistant signaling occurs, continued sub-lethal stresses could induce the accumulation of aberrant tumorigenic signaling and eventually cause the development and progression of cancers. It has been also reported that HO1 might be anti-tumorigenic by protecting healthy normal cells, but also protecting already established cancer [52]. Therefore, additional study is needed to elucidate the role of the NGF-HO1 pathway and its potential as a therapeutic target for human cancers.

## Conclusions

In conclusion, this study demonstrated that the expression of NGF and HO1 might be helpful for the estimation of prognosis of gastric carcinoma patients. In addition, this study suggests that regulation of the NGF-HO1 pathway might be a new therapeutic strategy for the treatment of gastric carcinoma patients with significant risk factors.

## Abbreviations

95% CI: 95% confidence interval
CA19-9: Carbohydrate antigen 19–9
CEA: Carcinoembryonic antigen
HO1: Heme oxygenase-1
NGF: Nerve growth factor
NGFR: Nerve growth factor receptor
OS: Overall survival
p75^NTR^: p75 neurotrophin receptor
PI3K: Phosphatidylinositol 3-kinase
proNGF: Precursor of NGF (proNGF)
RFS: Relapse-free survival
TMA: Tissue microarray
TrkA: Tropomyosin receptor kinase A
